# Circadian clock control of tRNA synthetases in *Neurospora crassa*

**DOI:** 10.12688/f1000research.125351.2

**Published:** 2023-08-17

**Authors:** Kathrina D. Castillo, Emily D. Chapa, Teresa M. Lamb, Madhusree Gangopadhyay, Deborah Bell-Pedersen

**Affiliations:** 1 Biology, Texas A&M University, College Station, TX, 77843, USA; 2 Center for Biological Clocks Research, Texas A&M University, College Station, TX, 77843, USA

**Keywords:** Circadian clock, Neurospora cras, tRNA synthetases, translation control

## Abstract

**Background:** In *Neurospora crassa*, the circadian clock controls rhythmic mRNA translation initiation through regulation of the eIF2α kinase CPC-3 (the homolog of yeast and mammalian GCN2). Active CPC-3 phosphorylates and inactivates eIF2α, leading to higher phosphorylated eIF2α (P-eIF2α) levels and reduced translation initiation during the subjective day. This daytime activation of CPC-3 is driven by its binding to uncharged tRNA, and uncharged tRNA levels peak during the day under control of the circadian clock. The daily rhythm in uncharged tRNA levels could arise from rhythmic amino acid levels or aminoacyl-tRNA synthetase (aaRSs) levels.

**Methods**: To determine if and how the clock potentially controls rhythms in aspartyl-tRNA synthetase (AspRS) and glutaminyl-tRNA synthetase (GlnRS), both observed to be rhythmic in circadian genomic datasets, transcriptional and translational fusions to luciferase were generated. These luciferase reporter fusions were examined in wild type (WT), clock mutant Δ *frq*, and clock-controlled transcription factor deletion strains.

**Results:** Translational and transcriptional fusions of AspRS and GlnRS to luciferase confirmed that their protein levels are clock-controlled with peak levels at night. Moreover, clock-controlled transcription factors NCU00275 and ADV-1 drive robust rhythmic protein expression of AspRS and GlnRS, respectively.

**Conclusions:** These data support a model whereby coordinate clock control of select aaRSs drives rhythms in uncharged tRNAs, leading to rhythmic CPC-3 activation, and rhythms in translation of specific mRNAs.

## Introduction

Aminoacyl-tRNA synthetases (aaRSs) play a fundamental role in mRNA translation by catalyzing the attachment of specific amino acids onto their cognate tRNAs. For accuracy, aaRSs employ chemical selectivity and proofreading capabilities ( Rubio Gomez and Ibba, 2020, Roux and Topisirovic, 2012). Mounting evidence supports that aaRSs have functions beyond their role in charging tRNAs, including roles in immune signaling, cell cycle, nutrient metabolism and growth, and thus are linked to various human diseases ( Pang *et al*., 2014, Nie *et al*., 2019, Park *et al*., 2008). Aminoacylation mechanisms are conserved across all kingdoms of life. The regulation of aaRS expression in prokaryotes is well-described ( Green *et al.*, 2010, Pelchat and Lapointe, 1999), and while less is known about their regulation in eukaryotes, coordinate induction of several mammalian cytoplasmic aaRS genes by amino acid starvation has been observed ( Shan *et al.*, 2016). In addition, several aaRSs were reported to have daily rhythms in abundance at the mRNA and/or protein levels in the filamentous fungus *Neurospora crassa* ( Sancar *et al*., 2015, Hurley *et al*., 2018, Castillo *et al*., 2022b), and mammalian cells ( Pembroke *et al*., 2015, Barclay *et al*., 2012, Hughes *et al*., 2009, Miller *et al*., 2007, Vollmers *et al*., 2012, Eckel-Mahan and Sassone-Corsi, 2009, Geyfman *et al*., 2012, Yoshitane *et al*., 2014, Janich *et al*., 2015). These data suggested that the circadian clock imparts regulation on *aars* gene expression, which would impact rhythmic protein synthesis and clock-controlled cellular processes.

The circadian clock is an endogenous timekeeping mechanism that regulates diverse biological processes in many organisms, allowing them to anticipate and prepare for daily environmental cycles, and to organize cellular processes to the right time of day for improved fitness ( Dunlap and Loros, 2017). Disruption of the circadian clock has profound effects on human physiology and behavior, and can lead to a wide range of diseases ( Bass, 2017, Foster, 2020, Hernandez-Garcia *et al*., 2020). Depending on the organism and tissue type, the circadian clock regulates daily rhythms in mRNA and protein accumulation for up to 50% of the eukaryotic genome ( Hurley *et al*., 2014, 2018, Zhang *et al*., 2014, Mauvoisin *et al*., 2014). Remarkably, most of the proteins that cycle in abundance under the control of the circadian clock are produced from mRNAs that are not clock-controlled ( Reddy *et al*., 2006, Robles *et al*., 2014, Zhang *et al*., 2014, Hurley *et al*., 2018, Castillo *et al*., 2022b). These data suggested a prominent role for clock regulation of post-transcriptional processes, including rhythmic mRNA translation.

The *N. crassa* circadian clock is composed of negative elements FREQUENCY (FRQ), FRQ-INTERACTING RNA HELICASE (FRH), and CASEIN KINASE 1 (CK1), and positive elements WHITE COLLAR-1 (WC-1) and WHITE COLLAR-2 (WC-2) ( Baker *et al*., 2012, Dunlap and Loros, 2017). WC-1 and WC-2 heterodimerize to form the White Collar Complex (WCC) which binds to the promoters of *frq* and downstream clock-controlled genes (ccgs), including 24 transcription factors, to drive their rhythmic transcription ( Froehlich *et al*., 2003, Smith *et al*., 2010). In addition, the *N. crassa* clock generates rhythms in the activities of the conserved eukaryotic translation initiation factor 2 (eIF2) and eukaryotic translation elongation factor 2 (e-EF2) ( Caster *et al*., 2016, Karki *et al*., 2020, Ding *et al*., 2021). A central mechanism for translational control is the phosphorylation of eIF2α, as even partial phosphorylation is sufficient to inhibit protein synthesis ( Baird and Wek, 2012). Furthermore, rhythms in activity of the *N. crassa* eIF2α kinase CPC-3, a homolog of the well-studied yeast and mammalian eIF2α kinase GCN2, are dependent on rhythmic uncharged tRNA ^Val^_,_ levels. The rhythm in uncharged tRNA ^Val^ levels is driven, at least in part, by rhythms in valyl-tRNA synthetase (ValRS) levels ( Karki *et al*., 2020). However, in addition to ValRS, several other aaRSs were found to be clock-controlled from genomic datasets ( Sancar *et al*., 2015, Hurley *et al*., 2018, Castillo *et al*., 2022b), suggesting that aaRSs may be coordinately regulated by the clock to control rhythmic translation.

In this study, we sought to independently validate clock control of two aaRSs and begin to examine the mechanisms of clock control of aaRS genes. Using aspartyl-tRNA synthetase (AspRS) and glutaminyl-tRNA synthetase (GlnRS) luciferase translational reporters, we confirmed that AspRS and GlnRS protein levels are rhythmic in WT cells with a peak in the subjective night, similar to the peak time of ValRS ( Karki *et al*., 2020), and arrhythmic in clock mutant Δ *frq* cells. We identified clock-controlled transcription factors that regulate AspRS::LUC and GlnRS::LUC rhythmic accumulation. We show that AspRS levels are low and arrhythmic in cells deleted for transcription factor NCU00275, and GlnRS levels are high and display a low amplitude rhythm in cells deleted for transcription factor ADV-1. These findings provide a basis for further studies investigating coordinate clock control of aaRSs via different clock-controlled transcription factors and the roles of rhythmic aaRS’s in rhythmic mRNA translation.

## Methods

### *N. crassa* strains and growth conditions

Strains, key reagents, and oligonucleotide primers used in this study are listed in Table 1. *N. crassa* wild type 74-OR23-IV (FGSC 4200) was grown in Vogel’s minimal media with 2% glucose (V2G) ( Davis and De Serres, 1970). All strains containing the hygromycin phosphotransferase ( *hph)* construct conferring resistance to hygromycin B were maintained on V2G and supplemented with 200 μg/mL of hygromycin B (VWR). Strains containing the *bar* cassette conferring resistance to Basta were maintained on V2G lacking NH _4_NO _3_ and supplemented with 0.5% proline (Sigma-Aldrich) and 200 μg/mL of Basta (Liberty ^TM^, Bayer).

**Table 1.** Key resources table.

Reagent or resource	Source	Identifier
**Plasmids**		
Plasmid pRMP57	Gooch *et al*., 2008	GenBank KC890770.1
Plasmid pBP15	Beasley *et al*., 2006	N/A
**Chemicals, peptides, and recombinant proteins**		
Basta (Liberty ^TM^)	Bayer	Cat#280SL
Hygromycin	VWR	Cat#80055-268
Luciferin	Gold Biotechnology	Cat#LUNCA-300
**Strains**		
*Neurospora crassa* wild type 74-OR23-IV mat a	FGSC	FGSC 4200; DBP 985
Δ *frq::bar*, mat A	Bennett *et al*., 2013	DBP 1228
Δ *ncu00275::hph*, mat a	FGSC	FGSC 12217; DBP 927
Δ *adv-1::hph*, mat A	FGSC	FGSC 11042; DBP 917
Δ *clr-1::hph*, mat a	FGSC	FGSC 11029; DBP 981
Δ *sah-1::hph*, mat a	FGSC	FGSC 11132; DBP 990
Δ *vos-1::hph*, mat a	FGSC	FGSC 13536; DBP 1970
WT, AspRS::LUC translational fusion	This paper	DBP 3999
WT, GlnRS::LUC translational fusion	This paper	DBP 3991
WT, GlnRS::LUC translational fusion	This paper	DBP 3992
Δ *frq::bar*, AspRS::LUC translational fusion	This paper	DBP 4000
Δ *frq::bar*, GlnRS::LUC translational fusion	This paper	DBP 3989
Δ *ncu00275:hph*, AspRS::LUC translational fusion	This paper	DBP 4142
Δ *clr-1::hph*, AspRS::LUC translational fusion	This paper	DBP 4137
Δ *ncu00275::hph*, GlnRS::LUC translational fusion	This paper	DBP 4211
Δ *adv-1::hph*, GlnRS::LUC translational fusion	This paper	DBP 4208
WT, P *asprs::luc* transcriptional fusion	This paper	DBP 4280
WT, P *glnrs::luc* transcriptional fusion	This paper	DBP 4283
WT, P *asprs::luc* transcriptional fusion	This paper	DBP 4416
∆ *frq::bar*, P *asprs::luc* transcriptional fusion	This paper	DBP 4419
WT, P *glnrs::luc* transcriptional fusion	This paper	DBP 4407
∆ *frq::bar,* P *glnrs::luc* transcriptional fusion	This paper	DBP 4411
**Oligonucleotides**		
To generate AspRS::LUC asprsF1 5′ CAAAGCAACATGGCCGACAG 3′	This paper	N/A
To generate AspRS::LUC asprsR1 5′ TGGCGTCCTCAGGAAGCAACCTCTTGGGCG 3′	This paper	N/A
To generate AspRS::LUC asprsF2 5′ GTTGCTTCCTGAGGACGCCAAGAACATCAA 3′	This paper	N/A
To generate AspRS::LUC asprsR2 5′ ATGAAGTCACTTAATCAGACGGCGATCTTG 3′	This paper	N/A
To generate AspRS::LUC asprsF3 5′ GTCTGATTAAGTGACTTCATTGTCGGTGGG 3′	This paper	N/A
To generate AspRS::LUC asprsR3 5′ GATAACTGAAGGCTCGAAAT 3′	This paper	N/A
Validation of endogenous integration of AspRS::LUC asprsF4 5′ GCGATGGCATGCTGCCGACG 3′	This paper	N/A
Validation of endogenous integration of AspRS::LUC asprsR4 5′ GTCAGCTTGCTTCCCATAAG 3′	This paper	N/A
To generate GlnRS::LUC glnrsF1 5′ CAACTGCTACCTCCGATTCG 3′	This paper	N/A
To generate GlnRS::LUC glnrsR1 5′ TGGCGTCCTCGTTCTTCTCCTTATCCTCCT 3′	This paper	N/A
To generate GlnRS::LUC glnrsF2 5′ GGAGAAGAACGAGGACGCCAAGAACATCAA 3′	This paper	N/A
To generate GlnRS::LUC glnrsR2 5′ GGACAACTGCTTAATCAGACGGCGATCTTG 3′	This paper	N/A
To generate GlnRS::LUC glnrsF3 5′ GTCTGATTAAGCAGTTGTCCGCGAATTAAC 3′	This paper	N/A
To generate GlnRS::LUC glnrsR3 5′ TAGAGGTAGTACTGCCAGCG 3′	This paper	N/A
Validation of endogenous integration of GlnRS::LUC glnrsF4 5′ TGAAGTTTGGTGATGTCTCC 3′	This paper	N/A
Validation of endogenous integration of GlnRS::LUC glnrsR4 5′ TCCGAATAGTACTTCTGTGG 3′	This paper	N/A
To generate P *asprs*::luc asprsF5 5′ ATCGGCGGCCGCACGGATTATACGATGCCCGC 3′	This paper	N/A
To generate P *asprs*::luc asprsR5 5′ CGATACTAGTGTTGCTTTGTCGAATTCGAT 3′	This paper	N/A
To generate P *glnrs*::luc glnrsF5 5′ ATCGGCGGCCGCGTATTGAAAATAGGTGGGGA 3′	This paper	N/A
To generate P *glnrs*::luc glnrsR5 5′ CGATACTAGTGATGTGTCTGTGTGTGTGGT 3′	This paper	N/A
**Software and algorithms**		
BioDare2 (beta version 2)	Zielinski *et al*., 2014	https://biodare2.ed.ac.uk/
Cosine Wave Analysis	Lamb *et al*., 2011	PRISM software package (GraphPad Software)
ECHO	De Los Santos *et al*., 2020	https://github.com/delosh653/ECHO
ggplot2 R package	Wickham, 2016	https://ggplot2.tidyverse.org
GraphPad Prism version 9.4.0		https://www.graphpad.com/
Serial Cloner version 2.6.1		http://serialbasics.free.fr/Serial_Cloner.html
**Other**		
EnVision Xcite Multilabel Reader	PerkinElmer	Cat#2105-0010
NanoDrop ^TM^ Microvolume Spectrophotometer	Thermo Scientific	Cat#ND-ONE-W

To assay rhythmic translation of aspartyl tRNA synthetase (AspRS, NCU00915) and glutaminyl tRNA synthetase (GlnRS, NCU07926), an aaRS::LUC translational fusion to luciferase was generated by 3-way fusion polymerase chain reaction (PCR). Primers RSF1 and RSR1 were used to make fragment 1, and primers RSF3 and RSR3 were used to make fragment 3, both using wild type (WT) genomic DNA as template. Primers RSF2 and RSR2 were used to make fragment 2 using pRMP57, a plasmid containing the *N. crassa* codon-optimized luciferase gene as template DNA ( Gooch *et al*., 2008). The three PCR fragments with overlapping regions were stitched via fusion PCR using primers RSF1 and RSR3, and the resulting PCR product was co-transformed with the plasmid pBP15 containing *hph* ( Beasley *et al*., 2006) into WT (FGSC2489). Primers were designed using Serial Cloner version 2.6.1 and the sequences are given in Table 1. Lyophilized PCR oligonucleotide primers were obtained from Integrated DNA Technologies (IDT) and resuspended in 1x Tris-EDTA, pH 8.0 buffer to make 100 μM primer stocks. The PCR reaction mix was as follows: total reaction mix = 50 μl, water = 37.5 μl, 5X High Fidelity (HF) buffer = 5 μl, dNTP mix = 1 μl (10 mM), primers = 2.5 μl each (10 μM), Phusion ^®^ DNA polymerase = 0.5 μl (1.0 units/50 μl PCR), and template DNA = 1 μl (50 ng). Phusion ^®^ High Fidelity DNA polymerase kit (Cat. No. M0530) and dNTP mix (Cat. No. 4030) were purchased from New England Biolabs (NEB) and Takara, respectively. The following annealing temperatures and extension times were applied: AspRS fragment 1 = 65°C, 1:30 min, fragment 2 = 72 ^o^C, 1:30 min, fragment 3 = 59°C, 1:30 min, and fragments 1+2+3 = 59°C, 3 min; GlnRS fragment 1 = 63°C, 1:30 min, fragment 2 = 72°C, 1:30 min, fragment 3 = 64 ^o^C, 30 s, and fragments 1+2+3 = 63°C, 3 min. PCR cycling was performed with a C1000 Touch Thermal Cycler (Bio-Rad) using the following program: 30 cycles of denaturation at 98°C for 10 s, annealing at varying temperatures for 30 s (as described above) and extension at 72°C for varying times (as described above).

Hygromycin-resistant transformants were screened for luciferase activity and homologous insertion into the *aars* gene (primers RSF4 and RSR4) using the same PCR conditions described above with annealing temperatures and extension times for AspRS = 62°C, 4 min, and GlnRS = 59°C, 4 min. To generate aaRS::LUC in different mutant strains, aaRS::LUC, WT were crossed with the knockouts on synthetic cross medium supplemented with 0.25% biotin ( Westergaard and Mitchell, 1947, Davis and De Serres, 1970). AspRS::LUC transformants were crossed with Δ *frq::bar* (DBP 1228) to generate AspRS::LUC, WT (DBP 3999), AspRS::LUC, Δ *frq::bar* (DBP 4000). GlnRS::LUC transformants were crossed with Δ *frq::bar* (DBP 1228) ( Bennett *et al*., 2013) to generate GlnRS::LUC, WT (DBP 3991 *mat A* and DBP 3992 *mat a*), GlnRS::LUC, Δ *frq::bar* (DBP 3989). DBP 3999 was crossed with Δ *clr-1::hph* (DBP 981) and Δ *ncu00275* (DBP 927) to generate AspRS::LUC, Δ *clr-1::hph* (DBP 4137) and AspRS::LUC, Δ *ncu00275::hph* (DBP 4142), respectively. DBP 3992 was crossed with Δ *adv-1::hph* (DBP 917) to generate GlnRS::LUC, Δ *adv-1::hph* (DBP 4137). DBP 3991 was crossed with Δ *ncu00275::hph* (DBP 927) to generate GlnRS::LUC, Δ *ncu00275::hph* (DBP 4211).

To generate transcriptional fusions to *luc*, promoter regions of *asprs* (primers asprsF5 and asprsR5 to generate the 1.8 kb fragment P *asprs*), and *glnrs* (primers glnrsF5 and glnrsR5 to generate the 1.82 kb fragment P *glnrs*) were amplified by PCR using the cycling conditions described above with annealing temperatures and extension times for fragments P *asprs* and P *glnrs* = 68 ^o^C, 1:30 min. PCR products were digested with *Not*I and *Spe*I (NEB), and cloned into plasmid pRMP57 containing the codon-optimized luciferase gene. The resulting plasmids were linearized by *Pci*I (NEB) digest, co-transformed with hyg ^R^ pBP15 into WT (FGSC 4200) cells, and hygromycin-resistant transformants were screened for luciferase activity. P *asprs::luc* transformants were crossed with Δ *frq::bar* (DBP 1228) ( Bennett *et al.*, 2013) to generate P *asprs::luc*, WT (DBP 4416) and P *asprs::luc*, Δ *frq::bar* (DBP 4419). P *glnrs::luc* transformants were crossed with Δ *frq::bar* (DBP 1228) to generate P *glnrs::luc*, WT (DBP 4407), and P *glnrs::luc*, Δ *frq::bar* (DBP 4411).

### Luciferase assays

To examine bioluminescence rhythms arising from strains containing luciferase fusions, 5 μl of a 1×10 ^5^ conidia/ml suspension were inoculated into 96 well microtiter plates containing 150 μl of 1X Vogel’s salts, 0.01% glucose, 0.03% arginine, 0.1 M quinic acid, 1.5% agar, and 25 μM firefly luciferin, pH 6. After inoculation of conidia (1×10 ^5^ conidia), the microtiter plate was incubated at 30°C in LL for 24 h and transferred to DD 25°C to obtain bioluminescence recordings using EnVision Xcite Multilabel Reader (Perkin-Elmer), with recordings taken every 90 min over 4–5 days. Raw reads were normalized to the mean to graph the data.

### Sequencing datasets

Rhythmic expression of *N. crassa* clock-controlled tRNA synthetases was determined using public ribosome profiling (ribo-seq) and RNA-seq datasets for WT and clock mutant Δ *frq* cells ( Castillo *et al.*, 2022b). Briefly, ribo-seq and RNA-seq were performed in parallel from cells grown in constant darkness (DD) in a circadian time course with 4-h resolution for two biological replicates.

### Statistical test for rhythmicity and analysis of circadian parameters

Rhythmic data from luciferase assays were fit to a sine wave or a line as previously described ( Lamb *et al*., 2011). Nonlinear regression to fit the rhythmic data to a sine wave (fitting period, phase, and amplitude) and a line (fitting slope and intercept), as well as Akaike’s information criteria tests to compare the fit of each data set to the 2 equations, were carried out using the Prism software package version 9.4.0. The p-values reflect the probability that, for instance, the sine wave fits the data better than a straight line. Error bars in all graphs represent the standard error of the mean (SEM) from independent experiments. Raw and normalized luciferase activity reads were analyzed for period, phase, and amplitude values using BioDare version 2 ( Zielinski *et al*., 2014). Heat maps were generated using the ggplot2 R package for genes with rhythmic ribosome protected fragments (RPF) counts in WT, and sorted according to increasing peak phase of the oscillation ( Wickham, 2016). ribosome protected fragments (RPF) levels are standardized within each gene (row) (Z-scores).

## Results

### AspRS and GlnRS protein levels are clock-controlled

**Figure 1.**
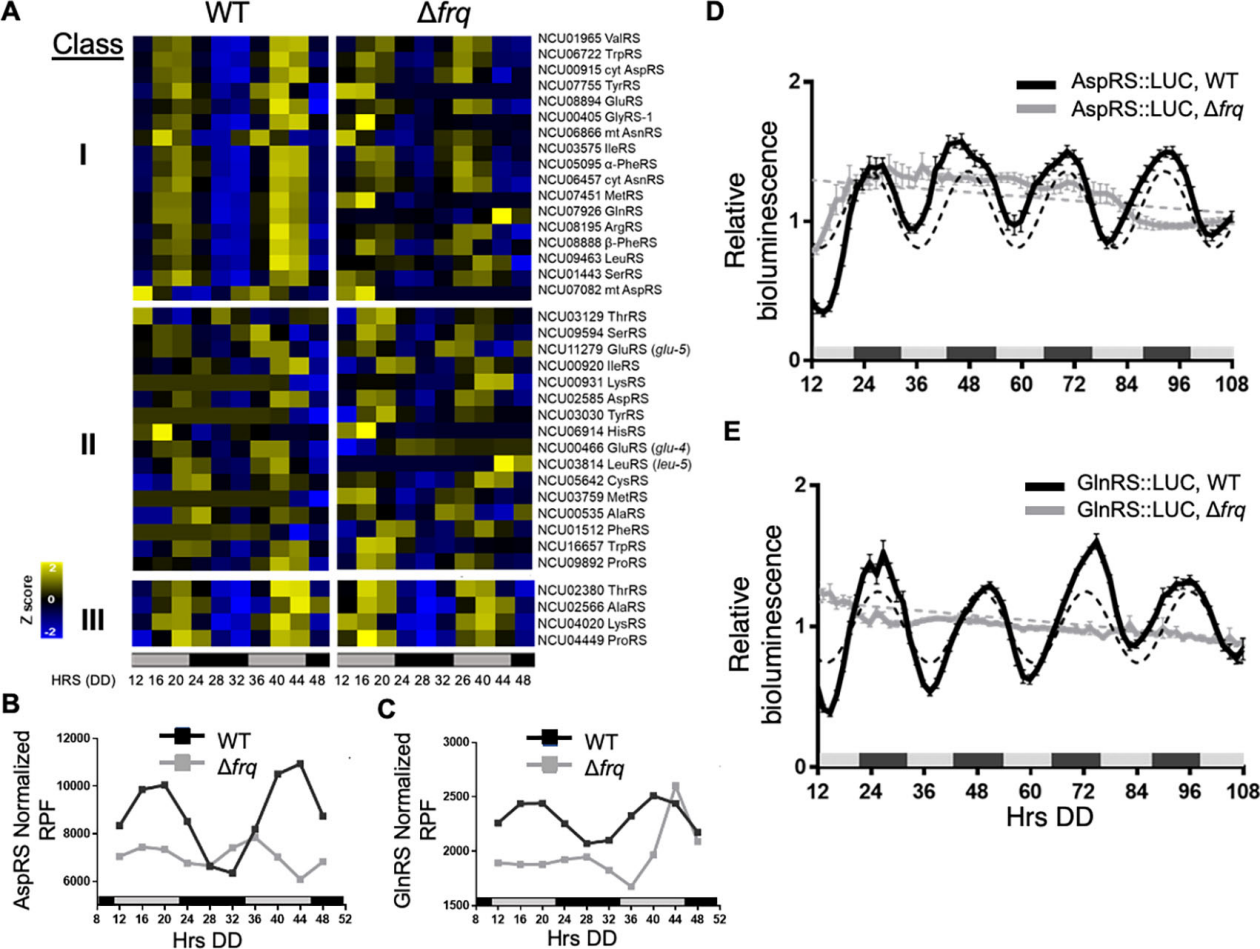
The circadian clock controls amino acid tRNA synthetase (aaRS) translational rhythms. A) Heat maps of the peak phase of *aaRS* mRNAs with rhythmic ribosome protected fragments (RPF) counts in WT, and arrhythmic RPF counts in Δ *frq* cells (Class I), arrhythmic in both WT and Δ *frq* cells (Class II), or rhythmic in both WT and Δ *frq* cells (Class III) from two replicate samples grown in DD and harvested at the indicated times (Hrs). Genes in Class I are sorted by the peak phase in WT. Cyt (cytoplasmic), mt (mitochondrial) B-C. Plots show the normalized fitted RPF reads (n=2) in WT (black line, ECHO p-value ≤ 0.05) and Δ *frq* (gray line, ECHO p-value > 0.05 for AspRS and p-value = 0.04 for GlnRS with a short 16 h period) cells for B) AspRS and C) GlnRS. Luciferase (LUC) activity from D) AspRS::LUC and E) GlnRS::LUC translational fusions in WT (black line) and Δ *frq* (gray line) cells. The average bioluminescence signal is plotted (AspRS::LUC, mean ± SEM, n=12 and GlnRS::LUC, mean ± SEM, n=24). Raw reads were normalized to the mean to plot the data. AspRS::LUC and GlnRS::LUC in WT cells were rhythmic as indicated by a better fit to a sine wave (dotted black line, p-value < 0.001). AspRS::LUC and GlnRS::LUC in Δ *frq* were arrhythmic as indicated by a better fit of the data to a line (dotted gray line p-value > 0.05). The bar at the bottom of the heat maps and graphs represents subjective day (gray) and subjective night (black) in this and all subsequent figures. Data for A-C from Castillo *et al.* (2022b).

Published *N. crassa c*ircadian ribosome profiling (ribo-seq) data revealed rhythms in ribosome occupancy for 17 of 36 aaRS using the Extended Circadian Harmonic Oscillator (ECHO) rhythmicity detection tool ( De Los Santos *et al*., 2020, Castillo *et al*., 2022b). Genes with an adjusted p-value of < 0.05, and with circadian harmonic, damped, or forced oscillation types were considered rhythmic. A heat map of the phase-sorted fitted ribosome protected footprint (RPF) values obtained using ECHO showed robust rhythmic ribosome occupancy for 17 aaRSs in WT cells, with peak ribosome occupancy primarily during the late subjective day (DD40-44) ( Figure 1A, Class I). As expected for circadian clock control, the rhythms were abolished in the clock mutant Δ *frq* cells. Class II aaRS genes were arrhythmic in both WT and Δ *frq* cells ( Figure 1A). Class III aaRS genes were rhythmic in both WT and Δ *frq* cells, suggesting that the rhythms are controlled by something other than the FRQ/WCC circadian oscillator ( Figure 1A). Circadian rhythms in ValRS protein levels were previously validated using a luciferase (LUC) translational reporter. ValRS::LUC levels peaked in the subjective night ( Karki *et al*., 2020), lagging the observed peak in ribosome occupancy ( Figure 1A).

In higher eukaryotes, 9 aaRSs form a multisynthetase complex (MSC) that is proposed to aid translation by providing a channel through which tRNAs can pass to reach bound aaRSs ( Hyeon *et al*., 2019). Interestingly, 5 of the 9 aaRSs in the complex (AspRS, GlnRS, GluRS, LeuRS, and MetRS) are clock-controlled based on our ribosome profiling datasets ( Figure 1A), and we focused on validating circadian clock control of AspRS and GlnRS ( Figure 1B & C). AspRS and GlnRS luciferase translational reporter fusions were generated (AspRS::LUC and GlnRS::LUC) and assayed for rhythmic luciferase levels from cells grown in DD over 4 days ( Figure 1D & C). Bioluminescence rhythms were observed for both AspRS::LUC and GlnRS::LUC, with peak levels during the early subjective night (e.g. DD 48) and a period of 22.4 ± 0.6 h and 22.0 ± 0.4 h, respectively ( Table 2). Similar to ValRS::LUC, the peak in AspRS::LUC and GlnRS::LUC levels occurred a few hours after the peak in ribosome occupancy ( Figure 1B- E). The AspRS::LUC and GlnRS::LUC rhythms were abolished in Δ *frq* cells, confirming that AspRS and GlnRS protein levels are clock-controlled ( Figure 1D & E, Table 2).

**Table 2.** Calculated periods and phases for AspRS::LUC, GlnRS::LUC (data from Figure 1), P *asprs::luc* and P *glnrs::luc* in WT cells (data from Figure 2) and AspRS::LUC or GlnRS::LUC in transcription factor knockout cells (data from Figures 3 & 4).

Strain	Period	Phase
AspRS::LUC, WT	22.4 ± 0.6	15.3 ± 2.8
AspRS::LUC, Δ *clr-1*	22.8 ± 0.6	13.5 ± 2.3
AspRS::LUC, Δ *ncu00275*	arrhythmic	arrhythmic
P *asprs::luc*, WT	22.2 ± 0.6	15.4 ± 1.7
P *asprs::luc*, Δ *frq*	arrhythmic	arrhythmic
GlnRS::LUC, WT	22.0 ± 0.4	14.8 ± 1.8
GlnRS::LUC, Δ *ncu00275*	23.8 ± 0.8	11.8 ± 2.2
GlnRS::LUC, Δ *adv-1*	22.5 ± 1.1, low amplitude	12.9 ± 3.3
P *glnrs::luc*, WT	22.0 ± 0.5	15.8 ± 1.7
P *glnrs::luc*, Δ *frq*	arrhythmic	arrhythmic

### Clock-controlled transcription factors drive rhythms in AspRS and GlnRS expression

**Figure 2.**
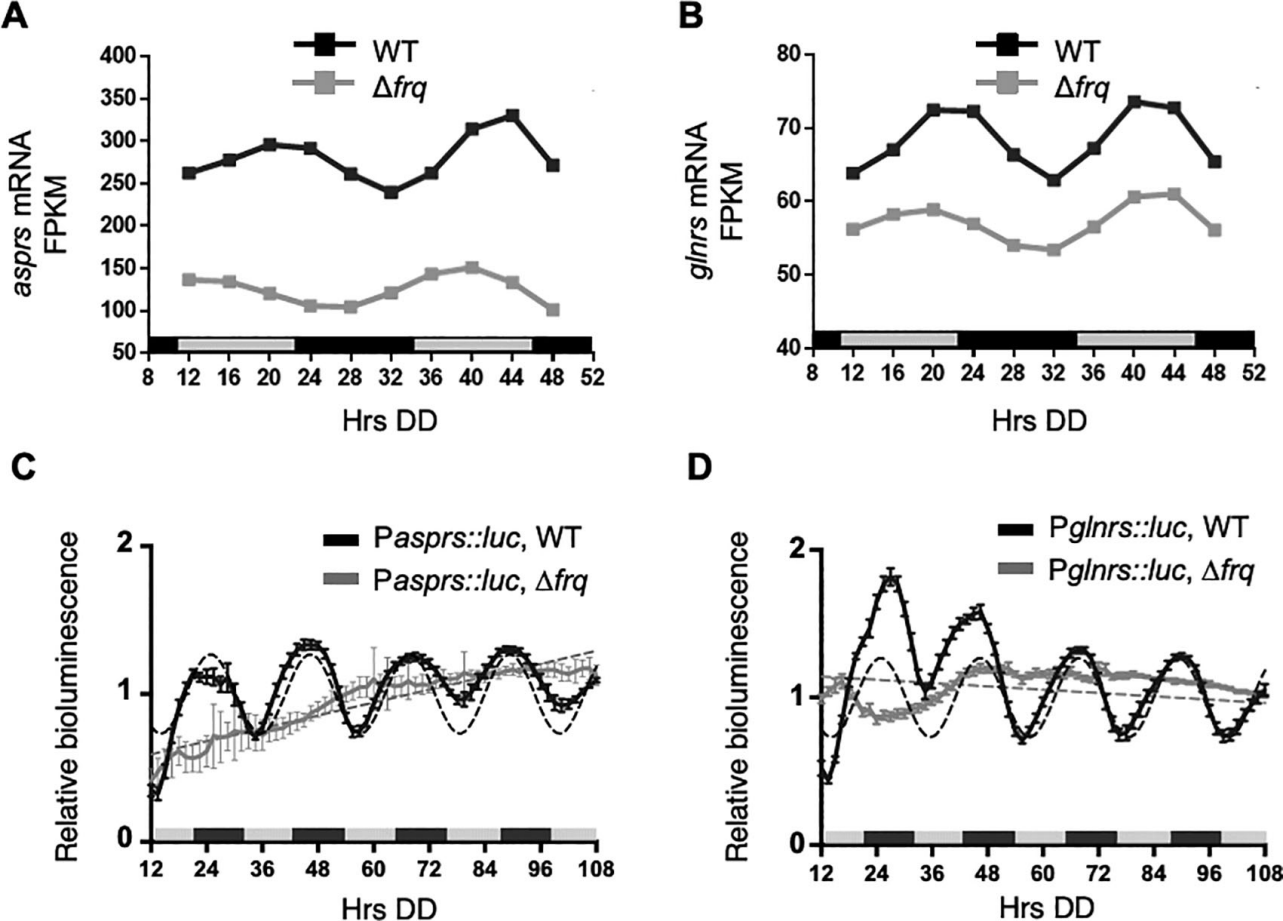
*asprs* and *glnrs* mRNA are clock-controlled. A-B. Plots show the mRNA fragments per kilobase of exon per million mapped fragments (FPKM) levels in WT (black line, ECHO p-value < 0.05) and Δ *frq* (gray line, ECHO p-value > 0.05) cells for A) *asprs* and B) *glnrs.* C-D. Plots show the luciferase activity from C) P *asprs::luc* transcriptional (black line) and D) P *glnrs::luc* transcriptional (black line) fusions in (black line) and *∆frq* (grey line) cells grown in DD and recorded every 90 min over 4 d (Hrs DD). The average bioluminescence signal is plotted (mean ± SEM, n=12). Luciferase activities are rhythmic as indicated by a better fit to a sine wave (dotted black line, p-value < 0.0001) and arrhythmic in *∆frq* cells where the best fit is to a line (dotted grey line). Data for A-B from Castillo *et al.* (2022b).

In addition to rhythms in protein levels, several *N. crassa* aaRS mRNAs were reported in genome-wide studies to be clock-controlled ( Hurley *et al*., 2014, 2018, Sancar *et al*., 2015, Castillo *et al*., 2022b). Of these aaRSs, *asprs* and *glnrs* exhibited rhythms in mRNA levels in WT cells, with mRNA levels peaking in the subjective early evening (DD40-44) ( Figure 2A & B). In support of these genomic data, *asprs* (P *asprs::luc*) and *glnrs* (P *glnrs::luc*) promoter *luc* fusions were rhythmic in DD peaking during the subjective night ( Figure 2C & D), with no significant period and phase differences between the mRNA and protein levels ( Table 2).

As expected for clock control, the mRNA rhythms were abolished in Δ *frq* cells as shown by the ECHO-generated fitted values for normalized mRNA levels by FPKM (fragments per kilobase of exon per million mapped reads) and by *aars* promoter luciferase reporter fusion assays ( Figure 2A & B) ( Castillo *et al*., 2022b). Together, these data support that rhythmic AspRS and GlnRS protein levels arise, at least in part, from clock-controlled mRNA levels.

**Figure 3.**
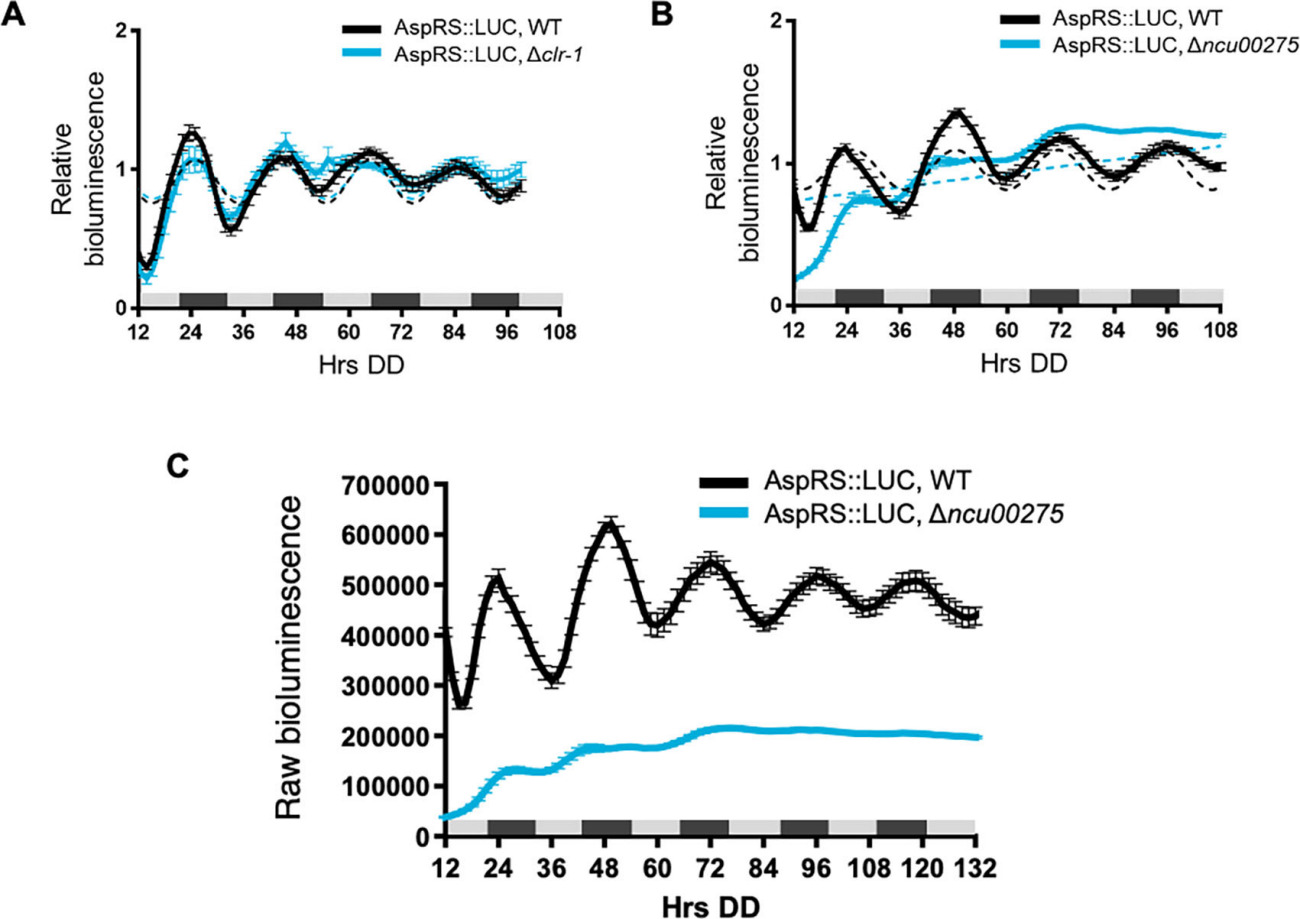
Transcription factor NCU00275 is required for robust rhythmic expression and AspRS protein levels. Luciferase activity from AspRS::LUC translational fusions in WT (black line) and transcription factor knockouts A) Δ *clr-1* (blue line) and B) Δ *ncu00275* (blue line). Raw reads were normalized to the mean to plot the data. The average bioluminescence signal is plotted (mean ± SEM, n=12). Luciferase activities are rhythmic as indicated by a better fit to a sine wave (dotted black line, p-value < 0.0001) or arrhythmic as indicated by a better fit of the data to a line (dotted blue line, p-value > 0.0001). C) Raw bioluminescence signals from AspRS::LUC translational fusions in WT (black line) and transcription factor knockout Δ *ncu00275* (blue line).

**Figure 4.**
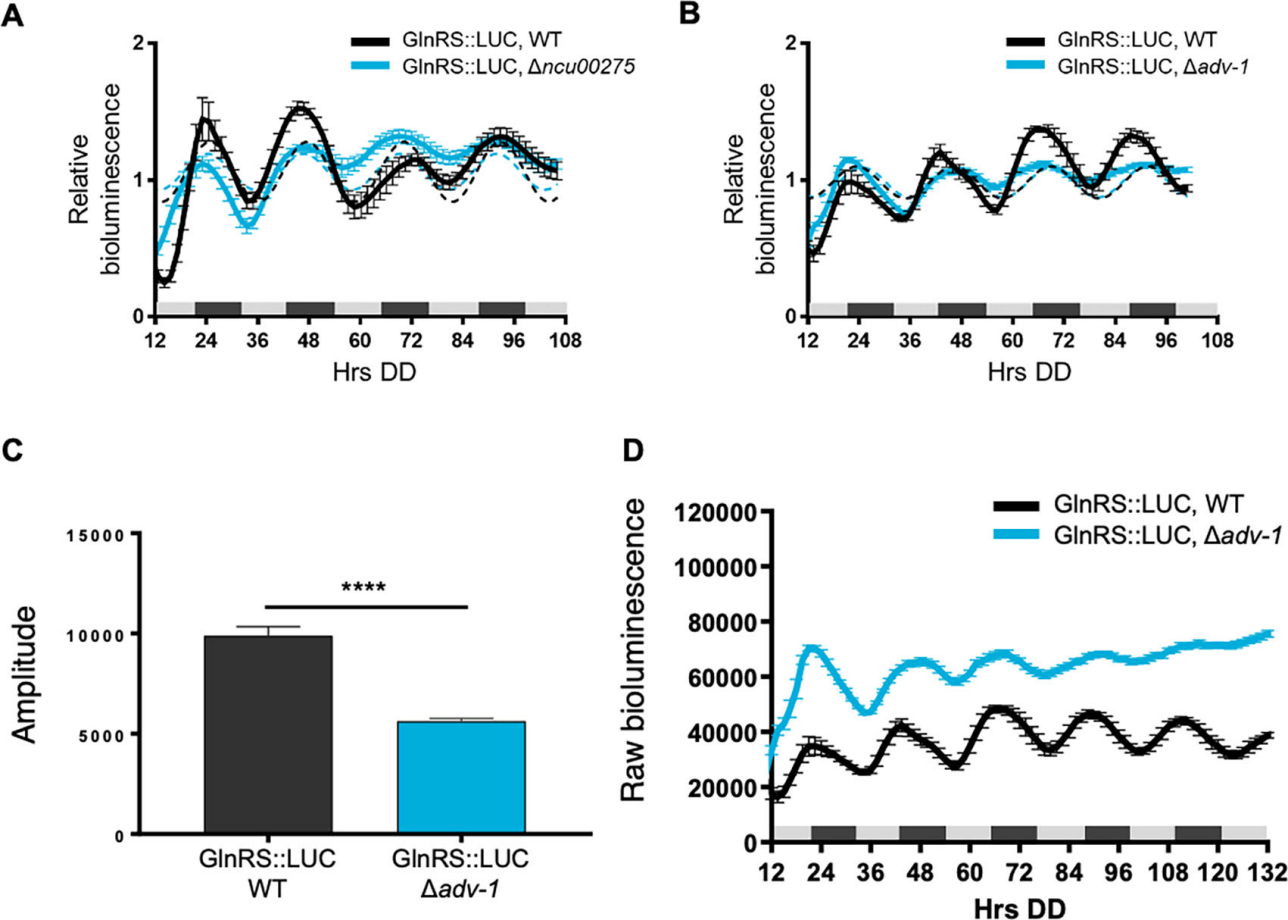
Transcription factor ADV-1 is required for robust rhythmic expression of GlnRS protein levels. Luciferase activities from GlnRS::LUC translational fusions in WT (black line) and transcription factor knockouts A) Δ *ncu00275* (blue line) and B) Δ *adv-1* (blue line). The average bioluminescence signal is plotted (mean±SEM, n=12). Luciferase activities are rhythmic as indicated by a better fit to a sine wave (dotted black line, p-value<0.0001) or arrhythmic as indicated by a better fit of the data to a line (dotted blue line, p-value>0.0001). C) Mean amplitude (mean ± SEM, n=12; ****p-value < 0.0001) of GlnRS::LUC bioluminescence traces in WT (black bar) and Δ *adv-1* (blue bar) cells. P-values were calculated by an unpaired *t*-test. D) Raw bioluminescence signals from GlnRS::LUC translational fusions in WT (black line) and transcription factor knockout Δ *adv-1* (blue line). The average bioluminescence signal is plotted (mean±SEM, n=12).

To determine if clock-controlled rhythms in AspRS and GlnRS protein levels require clock-controlled transcription factors and rhythmic transcription, we examined AspRS::LUC and GlnRS::LUC rhythms in cells deleted for transcription factors that are direct targets of the WCC. The levels of the transcription factor mRNAs peak in the subjective morning and the transcription factors bind to downstream ccgs to regulate their rhythmic expression ( Smith *et al*., 2010, Dekhang *et al*., 2017, Munoz-Guzman *et al*., 2021). Three clock-controlled transcription factors were selected for this initial study. CLR-1 (NCU07705) is a zinc binuclear cluster transcription factor that is important in nutrient sensing and signaling, and was shown to be necessary for normal AspRS mRNA levels when *N. crassa* cells were grown on cellulose ( Coradetti *et al.*, 2012). However, AspRS::LUC levels were rhythmic in Δ *clr-1* cells with a period and phase, and overall levels, that were similar to WT when the cultures were grown in DD ( Figure 3A, Table 2). Transcription factor NCU00275 was selected as a representative clock-controlled transcription factor of unknown function ( Smith *et al.*, 2010). AspRS::LUC rhythms were abolished in Δ *ncu00275* cells ( Figure 3B, Table 2) and AspRS::LUC levels were lower in Δ *ncu00275* compared to WT cells ( Figure 3C). These data supported that NCU00275 is necessary for circadian rhythms in AspRS::LUC levels and directly, or indirectly, activates *asprs* transcription. GlnRS::LUC was rhythmic in Δ *ncu00275* cells with no significant differences in levels, period or phase between WT and Δ *ncu00275* ( Figure 4A, Table 2). Previous RNA-seq data comparing WT to cells deleted for the transcription factor ADV-1 (NCU07392) showed that the levels of *glnrs* mRNA, but not *asprs* mRNA levels, were generally higher in Δ *adv-1* cells compared to WT grown in DD and following light treatments ( Dekhang *et al.*, 2017). ADV-1 ChIP-seq data did not reveal significant binding of ADV-1 to the promoter of *glnrs,* suggesting indirect negative regulation of *glnrs* by ADV-1. Based on these data, we examined GlnRS::LUC rhythms in Δ *adv-1*, and found that GlnRS::LUC rhythms became progressively damped by day 3 in DD compared to WT ( Figure 4B), leading to a significantly reduced amplitude of oscillation ( Figure 4C, Table 2). Consistent with the RNA-seq data, the raw bioluminescence signals showed that GlnRS::LUC levels were higher in Δ *adv-1* than in WT cells ( Figure 4D). Taken together, these data support that specific clock-controlled transcription factors contribute to the rhythmic expression of aaRSs.

All underlying data can be found in the *Underlying data* section ( Castillo *et al.*, 2022b, Castillo and Bell-Pedersen, 2022).

## Discussion

aaRSs play a central role in translation and translation fidelity, yet the regulation of aaRS gene expression in eukaryotes is understudied. Genome-wide datasets revealed that several eukaryotic aaRSs are clock-regulated at the level of mRNA and protein ( Sancar *et al*., 2015, Hurley *et al*., 2018, Castillo *et al*., 2022b, Barclay *et al*., 2012, Pembroke *et al*., 2015, Hughes *et al*., 2009, Miller *et al*., 2007, Vollmers *et al*., 2012, Eckel-Mahan and Sassone-Corsi, 2009, Geyfman *et al*., 2012, Yoshitane *et al*., 2014, Janich *et al*., 2015). We previously showed that the levels of ValRS cycle under control of the circadian clock with peak levels during the subjective night ( Karki *et al*., 2020). Here, we validated that *asprs* and *glnrs* mRNA and protein levels are also clock-controlled with a similar night-time peak in protein levels. The bulk of rhythmic protein accumulation occurs at night in *N. crassa* ( Hurley *et al*., 2018) supporting that the night-time peak in aaRS protein levels serve to coordinately increase protein synthesis at night.

We observed that AspRS and GlnRS protein rhythms are dependent on the circadian clock through the activities of clock-controlled transcription factors. AspRS levels are arrhythmic and low in Δ *ncu00275*, suggesting that NCU00275 activates *asprs* transcription. NCU00275 is annotated as a hypothetical protein, but its homologs in other fungi suggest that it is a C3HC4-type RING finger protein involved in transcription, signal transduction, ubiquitination, and recombination ( Basenko *et al*., 2018, Krishna *et al*., 2003). NCU00275 mRNA levels peak in the subjective morning under control of the WCC ( Smith *et al.*, 2010), whereas *asprs* mRNA and protein levels peak several hours later in the early subjective night. This delay suggests the possibility that the effect of NCU00275 on *asprs* transcription is indirect, requires other transcription factors or changes in chromatin state, and/or that posttranscriptional regulation of *asprs* contributes to this delay. Furthermore, deletion of NCU00275 had no significant effect on GlnRS levels, indicating that coordinate regulation of the aaRS’s by the clock is not necessarily through the same clock-controlled transcription factors. Consistent with this idea, the clock-controlled transcription factor ADV-1 was previously shown to affect the levels of *glnrs*, but not *asprs*, mRNA levels ( Dekhang *et al.*, 2017), and GlnRS levels and rhythms were altered in Δ *adv-1* cells compared to WT. Furthermore, ChIP-seq data showed that ADV-1 binds to the promoter of TrpRS (NCU06722) and MetRS (NCU07451), both of which are clock-controlled, and to the promoter of LysRS ( Dekhang *et al.*, 2017), which was not rhythmic in our ribo-seq data ( Figure 1). Additional experiments will be done in the future to confirm clock control of the other *N. crassa* aaRS’s predicted to be rhythmic from RNA-seq and ribo-seq datasets, and to comprehensively define the roles of clock-controlled transcription factors in aaRS regulation.

Charging tRNAs with the correct amino acid is the first step in translation, and therefore the levels and function of aaRSs are critical to translation fidelity ( Yu *et al*., 2021, Hausmann and Ibba, 2008). Mistakes in translation are generally considered detrimental; however, during stress, mistranslation may be beneficial by increasing the levels of altered proteins that can perform new functions to aid the response ( Pan, 2013, Ribas de Pouplana *et al*., 2014). In eukaryotes, some aaRSs form the MSC ( Bandyopadhyay and Deutscher, 1971, Lee *et al*., 2004, Kerjan *et al*., 1994), with varying composition dependent on the organism. In *S. cerevisiae*, the MSC is comprised of MetRS, GluRS and the scaffold protein Arc1 ( Galani *et al*., 2001). The MSC in mammals has 9 aaRSs, including 5 of the 9 aaRSs (AspRS, GlnRS, GluRS, LeuRS, and MetRS) that are rhythmic in ribo-seq data sets, and 3 scaffold proteins, AIMP1-3. The MSC helps the function of its components; for example, the K _m_ for binding of tRNA ^Met^ to MetRS in the yeast MSC is about 100-fold lower compared to the K _m_ for binding of tRNA ^Met^ to MetRS alone ( Simos *et al*., 1998). MSC components are also involved in cell signaling, stress responses, metabolite sensing, and controlling gene expression by binding to specific RNA and DNA sites, supporting the idea that a key role of the MSC is to support alternative functions of aaRSs ( Cui *et al*., 2021, Pang *et al*., 2014, Rubio Gomez and Ibba, 2020). Furthermore, aaRSs, either alone or in the MSC complex, participate in a wide variety of processes outside of their classical role in tRNA charging, including transcription regulation, splicing, and metabolism ( Rubio Gomez and Ibba, 2020), and abnormal expression, localization, and molecular interactions of aaRSs are associated with a variety of human diseases, including cancer ( Zhou *et al*., 2020). This widespread impact of aaRSs on host biology raises the intriguing idea that daily rhythms in the levels of aaRSs represent a missing factor linking the clock to a wide range of rhythmic biological processes that are critical to health, underscoring the need to better understand the mechanisms underlying their circadian regulation.

## Data availability

### Underlying data

Gene Expression Omnibus: Ribosome profiling and RNA-seq data used in Figures 1 and 2. Accession number GSE181566; https://identifiers.org/geo:GSE181566 ( Castillo *et al.*, 2022b).

Figshare: Circadian Clock Control of tRNA synthetases in *Neurospora crassa.* https://doi.org/10.6084/m9.figshare.c.6209830.v4 ( Castillo and Bell-Pedersen, 2022).

Data are available under the terms of the Creative Commons Attribution 4.0 International license (CC-BY 4.0).
