# Supplementary material for: Circadian clock control of tRNA synthetases in Neurospora crassa
Source: F1000Res. 2023 Aug 17;11:1556. Originally published 2022 Dec 22. [Version 2] doi: 10.12688/f1000research.125351.2 (PMC10576190; doi:10.12688/f1000research.125351.2)
Supplement: Peer review history

# Reviewer report: Reviewer response for version 2

**DOI:** 10.5256/f1000research.153682.r197684

**Published:** 2023-10-13

**Authors:** Luis F. Larrondo

**Affiliations:** 1 Millennium Science Initiative, Millennium Institute for Integrative Biology (iBio), Santiago, Chile

This reviewer appreciates the efforts made by the authors to address the prior points.

 It is surprising to see that the Class III aaRS show rhythms in *D*frq, which are overall just like WT (not amplitude nor phase appear to be affected).

 Hopefully the authors will tackle some of those aaRS in future studies. 

 One minor comment:

 Figure 2L (black line, ECHO p-value < 0.05) and Δ *frq* (gray line, ECHO p-value > 0.05).

 Could the authors indicate the p value for Dfrq, as visual inspection of the data shows a “:hint” of rhythmicity and therefore it would be informative to know if p value is 0.06 or 0.6  for example)

Is the work clearly and accurately presented and does it cite the current literature?

Yes

If applicable, is the statistical analysis and its interpretation appropriate?

Partly

Are all the source data underlying the results available to ensure full reproducibility?

Yes

Is the study design appropriate and is the work technically sound?

Yes

Are the conclusions drawn adequately supported by the results?

Partly

Are sufficient details of methods and analysis provided to allow replication by others?

Yes

Reviewer Expertise:

Circadian clocks  Neurospora biology

I confirm that I have read this submission and believe that I have an appropriate level of expertise to confirm that it is of an acceptable scientific standard.

# Reviewer report: Reviewer response for version 2

**DOI:** 10.5256/f1000research.153682.r197683

**Published:** 2023-08-24

**Authors:** Ruifeng Cao

**Affiliations:** 1 Department of Neuroscience and Cell Biology, Rutgers University New Brunswick, Piscataway, New Jersey, USA

All my comments were satisfactorily addressed.

Is the work clearly and accurately presented and does it cite the current literature?

Yes

If applicable, is the statistical analysis and its interpretation appropriate?

Yes

Are all the source data underlying the results available to ensure full reproducibility?

Yes

Is the study design appropriate and is the work technically sound?

Yes

Are the conclusions drawn adequately supported by the results?

Yes

Are sufficient details of methods and analysis provided to allow replication by others?

Partly

Reviewer Expertise:

circadian rhythm, translational control, neuroscience

I confirm that I have read this submission and believe that I have an appropriate level of expertise to confirm that it is of an acceptable scientific standard.

# Reviewer report: Reviewer response for version 1

**DOI:** 10.5256/f1000research.137649.r160525

**Published:** 2023-03-14

**Authors:** Luis F. Larrondo

**Affiliations:** 1 Millennium Science Initiative, Millennium Institute for Integrative Biology (iBio), Santiago, Chile

The work by Castillo et al., provides new information on how the circadian clocks impact cellular proteostasis, particularly how several tRNA synthases are rhythmically expressed. The observations contained in the MS provide a mechanistic perspective on the prior events leading to rhythmic translation via eIF2a.

 Overall the MS is nicely written and easy to follow. There are some minor points that could by further clarified.

1. “While the regulation of aaRS expression in prokaryotes is well-described, less is known about their regulation in eukaryotes (Frugier and Giege, 2003)”.
  Since this is a 20-year old ref, please provide additional references that exemplify that up to this date there is a knowledge gap in eukaryotes.
2. Table 2 legend fails to mention that it also compiles data extracted from Fig. 4.
3. In Fig 2. It appears that both tRNA synthase genes show some residual rhythm in the delta *frq* strain. Moreover, when one goes to the heatmap on Fig1, one may have a similar “impression”. Could the authors further discuss this and, particularly, comment if the rhythmicity indexes obtained with the software yielded borderline rhythmicity?
4. “We found, for example, that AspRS::LUC was rhythmic in WT and Δclr-1 (NCU07705) cells”.
  It is unclear why the authors decided to examine these two particular KOs (NCU07705 and NCU00275) in AspRS expression. Was the choice guided by bioinformatic analyses or literature curation?  The same applies to the GlnRS.
5. “Furthermore, the progressive dampening of GlnRS::LUC levels in Δadv-1 significantly reduced the amplitude of oscillation, leading to arrhythmicity (Figure 4D).”
  While I agree that the absence of adv-1 has an impact on GlnRS expression, I am not so convinced that the end result is arrhythmic gene expression.
  Eye-examination of Fig 4c reveals a distinct oscillatory pattern. I would even predict that if the rhythmicity analysis is limited to the first 72 or 96 h, the software will confirm circadian rhythms. Moreover, if one were to cross such strain with a frq7 allele, my prediction is that the expression of GlnRS in the absence of adv-1 will look indistinctively circadian (particularly aided by the overall higher amplitude of rhythms in frq7) and with a longer period.
6. “ GlnRS rhythms were less robust in Δadv-1”
  In the discussion, the authors are less categoric about the effect of adv-1 and don’t talk about arrhythmicity, while in table 2(and Fig 4) they use the latter concept.

Is the work clearly and accurately presented and does it cite the current literature?

Yes

If applicable, is the statistical analysis and its interpretation appropriate?

Partly

Are all the source data underlying the results available to ensure full reproducibility?

Yes

Is the study design appropriate and is the work technically sound?

Yes

Are the conclusions drawn adequately supported by the results?

Partly

Are sufficient details of methods and analysis provided to allow replication by others?

Yes

Reviewer Expertise:

Circadian rhythms, Neurospora genetics and gene expression

I confirm that I have read this submission and believe that I have an appropriate level of expertise to confirm that it is of an acceptable scientific standard, however I have significant reservations, as outlined above.

# Reviewer report: Reviewer response for version 1

**DOI:** 10.5256/f1000research.137649.r159010

**Published:** 2023-01-17

**Authors:** Ruifeng Cao

**Affiliations:** 1 Department of Neuroscience and Cell Biology, Rutgers University New Brunswick, Piscataway, New Jersey, USA

In the manuscript, Castillo et al. report circadian regulation of aminoacyl-tRNA synthetases (aaRS) in *Neurospora crassa*. The study started with genome-wide translational profiling around two circadian cycles and found 17 of 26 aaRS exhibited circadian rhythmicity in ribosomal occupancy. Next, they focused on two of the 17 rhythmic aaRS, AspRS and GlnRS. They found that their protein and mRNA levels were regulated by the circadian clock, and by clock-controlled transcription factors, e.g., NCU00275 and ADV-1. They conclude that select aaRS drive rhythms in uncharged tRNAs, which in turn leads to rhythmic CPC-3 activation and mRNA translation. Overall, this is an elegant study that nicely follows up with their previous work, and most results are compelling. The new findings will advance our understanding of translational control mechanisms by aaRS in the circadian clock. I have some moderate to minor comments to help improve the manuscript.

  

1. In Fig.1A, it would be informative and comprehensive if the heatmaps of the 19 aaRS that did not exhibit circadian rhythms in ribosome occupancy are also shown in the figure or in a supplementary figure.
2. In Fig.1 legend, RFP should be RPF (ribosome-protected fragments or footprints).
3. The Ribo-Seq experiment and data analysis were not described in Methods. What are the numbers of biological replicates for WT and Δfrq groups, respectively? N=17 is for which group? Are those 17 samples pooled or processed separately for Ribo-Seq? What are the sample sizes in Figs.1B, 1C, 2A, and 2B?
4. In all figures, the lengths of the bars indicating circadian day and night are slightly shorter than 24h. Are these determined based on the period data in Tab.2? 
5. Related to Figs. 2C and D, do P *asprs*::luc and P *glnrs*::luc exhibit rhythms in Δfrq cells? It would be interesting to see these data since the data in A and B show weak (although not statistically significant) rhythms in asprs and glnrs mRNA levels in Δfrq cells.
6. For audiences who are not familiar with the Neurospora clock, it would be helpful if more background information can be given regarding why the mutants Δclr-1 and Δncu00275 were selected in the current study. Please also discuss potential mechanisms for why AspRS::LUC was repressed and arrhythmic in Δncu00275 cells but the rhythms of GlnRS::LUC were barely affected. Are there any data indicating ncu00275 directly regulates asprs transcription?

Is the work clearly and accurately presented and does it cite the current literature?

Yes

If applicable, is the statistical analysis and its interpretation appropriate?

Yes

Are all the source data underlying the results available to ensure full reproducibility?

Yes

Is the study design appropriate and is the work technically sound?

Yes

Are the conclusions drawn adequately supported by the results?

Yes

Are sufficient details of methods and analysis provided to allow replication by others?

Partly

Reviewer Expertise:

circadian rhythm, translational control, neuroscience

I confirm that I have read this submission and believe that I have an appropriate level of expertise to confirm that it is of an acceptable scientific standard, however I have significant reservations, as outlined above.
